# Local management and landscape structure determine the assemblage patterns of spiders in vegetable fields

**DOI:** 10.1038/s41598-020-71888-w

**Published:** 2020-09-15

**Authors:** Hafiz Sohaib Ahmed Saqib, Junhui Chen, Wei Chen, Gabor Pozsgai, Komivi Senyo Akutse, Muhammad Furqan Ashraf, Minsheng You, Geoff M. Gurr

**Affiliations:** 1 State Key Laboratory of Ecological Pest Control for Fujian and Taiwan Crops, Fujian Agriculture and Forestry University, Fuzhou, 350002 China; 2 Joint International Research Laboratory of Ecological Pest Control, Ministry of Education, Fuzhou, 350002 China; 3 Institute of Applied Ecology, Fujian Agriculture and Forestry University, Fuzhou, 350002 China; 4 Key Laboratory of Integrated Pest Management for Fujian-Taiwan Crops, Ministry of Agriculture, Fuzhou, 350002 China; 5 Plant Health Division, International Centre of Insect Physiology and Ecology (ICIPE), P.O. Box 30772-00100, Nairobi, Kenya; 6 College of Crop Science, Fujian Agriculture and Forestry University, Fuzhou, 350002 China; 7 Graham Centre, Charles Sturt University, Orange, NSW 2800 Australia

**Keywords:** Agroecology, Biodiversity, Community ecology, Ecosystem services

## Abstract

Both field- and landscape-scale factors can influence the predator communities of agricultural pests, but the relative importance and interactions between these scales are poorly understood. Focusing on spiders, an important taxon for providing biological control, we tested the influence of field- and landscape-scale factors on structuring the spider communities in a highly dynamic brassica agroecosystem. We found that local factors (pesticide-use and crop type) and forested landscape significantly influenced the abundance and species richness of spiders, whilst grassland patches significantly affected the spider species richness. Correlation results demonstrated that assemblage patterns of most spider families positively responded to the interplay between local factors and forest patches in the landscape. The spiders abundance was greatest in cauliflower crops surrounded with forest and grassland patches in landscape. Similarly, ordination analyses revealed that organic fields of cauliflower in forested landscapes had a strong positive association with the abundance and species richness of spiders. In contrast, insecticide and synthetic fertilizer-treated fields of Chinese cabbage in landscapes with little non-crop habitat reduced the abundance and species richness of spiders. Our results highlight the extent of interaction between local- and landscape-scale factors, help explain recently reported inconsistent effects of landscape factors on conservation biological control.

## Introduction

Landscape features, together with chemical use practices, are known to influence the structure of ecological communities in agricultural systems^1–3^. As agricultural intensification increases, landscapes are structurally simplified, potentially leaving inadequate amounts of natural and semi-natural areas as refuge and donor habitat for invertebrates, including important ecosystem service providers^4^. Furthermore, intensive farming systems are also associated with increased pesticide use, and, as a consequence, the development of pesticide resistance^5,6^, the erosion of biodiversity and a loss of related ecosystem services including biological control of pests^7,8^. Organic and integrated pest management regimes, with lower pesticide use, can limit the loss of biodiversity, providing stronger ecosystem services to support agroecosystem productivity^9^.

A range of studies has reported the positive effects of the proportion of organic farming in the landscape on the abundance and species richness of beneficial arthropods^10,11^. Furthermore, in a conventional farming system, a multi-country study showed that strategic enhancement of biodiversity suppressed pest densities, and allowed a greatly reduced pesticide use with a parallel increase in yield^12^. Illustrating the potential link between local management and landscape-scale factors, arthropod communities in conventional farming systems depend heavily on the presence of nearby source habitats that allow recolonization in crops after pesticide use and associated episodic extermination of natural enemies within crops^13,14^. Therefore, there is a need to investigate the relative importance of local factors and wider landscape structures together in shaping the natural enemies’ community.

Structurally complex landscapes exert positive effects on the species abundance and richness patterns of arthropods from medium to large scales, extending to several kilometers from a focal field^1,15^. However, landscape complexity has several facets and the relative importance of each is not completely understood. At a smaller scale, adjacent crops or non-crop vegetation have been shown to act as a refuge for arthropods during times of adverse conditions or disturbance within a focal crop^16,17^. Thus, the arthropod community structures of natural enemies and crop pests can be affected by the availability and type of adjacent habitats^16,18^. For example, Saqib et al.^17^ demonstrated that certain forms of adjacent perennial crop and non-crop vegetation types benefited predatory spiders in brassica fields, most likely by serving as source habitats. On the other hand, Perović et al*.*^19^ showed the importance of the diversity of land use types and the spatial arrangement (including connectivity) of land uses up to 3 km from the focal field affected pest and natural enemies. Globally, a recent analysis of an especially large data set found that the presence of natural vegetation (woodland, grassland and scrubland) in the landscape led to inconsistent responses by pests and their predators and unpredictable yield outcomes^20^. This inconsistency suggests that local management factors, such as agronomic practices and inherent properties of crop types may also be important indirectly driving arthropod assemblages and mediating the effects of the wider landscape. Understanding the interplay between these wider landscape scales and local management practices is becoming important in highly disturbed and ephemeral cropping systems such as Brassica vegetables that have short-duration crop cycles, frequent tillage and often intense agrochemical use.

Spiders are an important group of ecosystem service providers, acting as natural enemies of pests^21^. They attack diverse groups of prey including pests such as aphids^22^ and lepidopteran larvae^23^. The effectiveness of the biological control of these generalist predators is strongly influenced by the structure and composition of their communities^24^. Previous studies have reported that landscape and local scale factors influenced the assemblages of diverse natural enemies in stable and less disturbed agroecosystems including grasslands^25^, pastures^26^, olive crop^27^, wheat fields^28^, cotton crop^19^ and vineyards^29^. But to the best of our knowledge, the present study represents the first attempt to shed light on how spider assemblages are shaped in a highly disturbed and ephemeral ecosystem, featuring different local field management factors, such as chemical use and crop types, and differing landscape structure. We hypothesized that spider density and diversity would be determined by local factors but mediated by the surrounding landscape composition, including proportion of different land uses.

## Results

### Spider families

In total, 2,809 individual spiders, representing 141 morphospecies, 63 molecularly identified species and 40 genera across 12 families were collected. When morphospecies were initially assigned, small visual differences including potential sexual dimorphisms, were considered potentially important, hence the lower number of molecularly confirmed species. Lycosidae was the dominant family (82.73% of individuals), followed by Theridiidae (4.63%), Salticidae (4.03%), Tetragnathidae (3.99%) and Linyphiidae (2.39%; see Supplementary dataset 1 for the complete list of species).

### Effects of field scale variables on spiders

**Figure 1:**
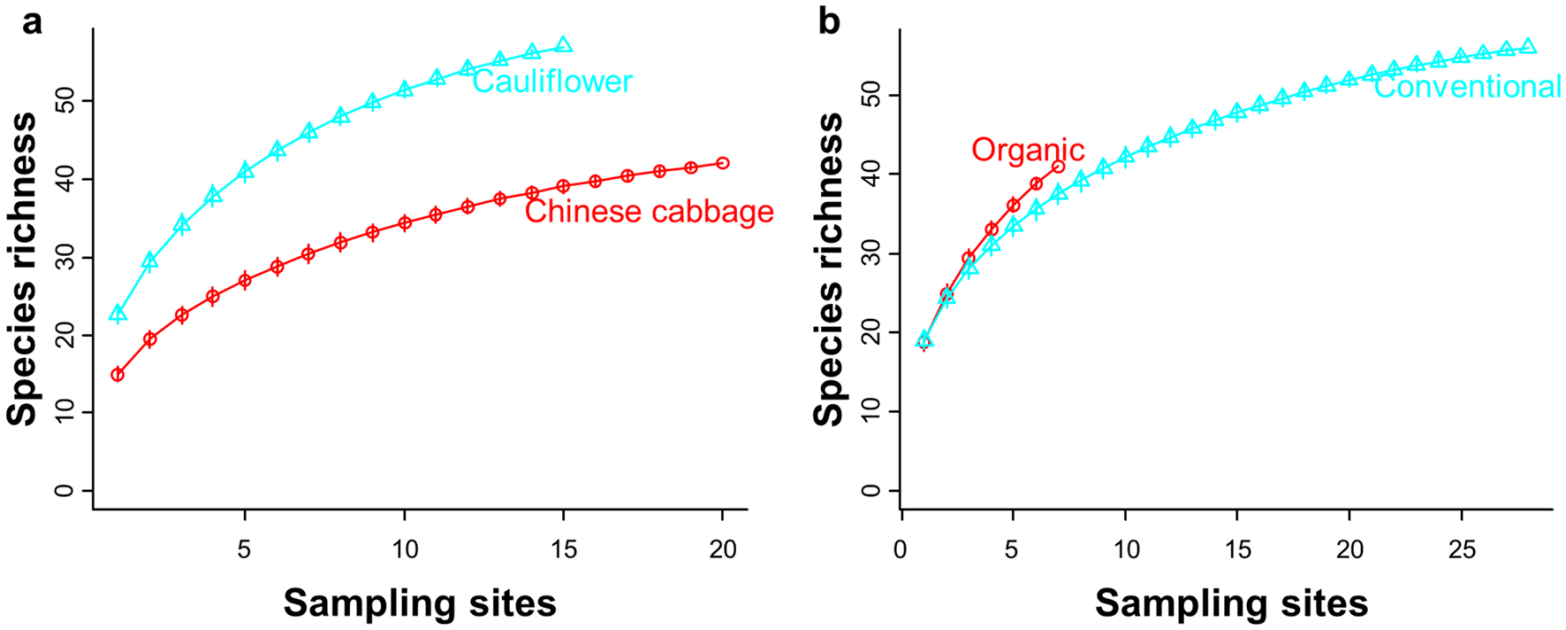
Species accumulation curves correspond to different taxa of spider samples collected from (**a**) two Brassica crops types under (**b**) the organic and conventional management practices. Curves represent the randomized samples.

Species accumulation curves showed a generally greater richness in cauliflower than in Chinese cabbage, with only Chinese cabbage reaching the asymptote (Fig. 1a). The spider species accumulation rate was increased more steeply across all organic sites compared to conventional sites, and further sampling is inferred to reveal greater species richness of spiders in organic than conventional fields (Fig. 1b).

Kruskal–Wallis test for all alpha diversity measures, (including richness, Shannon and Simpson indices) displayed significant (χ^2^ = 10.637, *p* = 0.001; χ^2^ = 8.801, *p* = 0.003 and χ^2^ = 6.760, *p* = 0.009, respectively) differences between spider assemblages in different Brassica crop types. In contrast, none of the alpha diversity measures differed significantly between organic and conventional fields (χ^2^ = 0.438, *p* = 0.508; χ^2^ = 0.980, *p* = 0.322 and χ^2^ = 0.979, *p* = 0.322, respectively). ANOSIM results revealed significant differences in spider assemblages in different crop types, both when family abundances and species richnesses were considered (R = 0.184, *p* = 0.001 and R = 0.182, *p* = 0.004, respectively). In contrast, no significant differences were observed between organic and conventional fields, if the family assemblage matrix was based on abundance (R = 0.067, *p* = 0.261) or species richness (R = 0.027, *p* = 0.371).

### Local and landscape interactions

**Figure 2:**
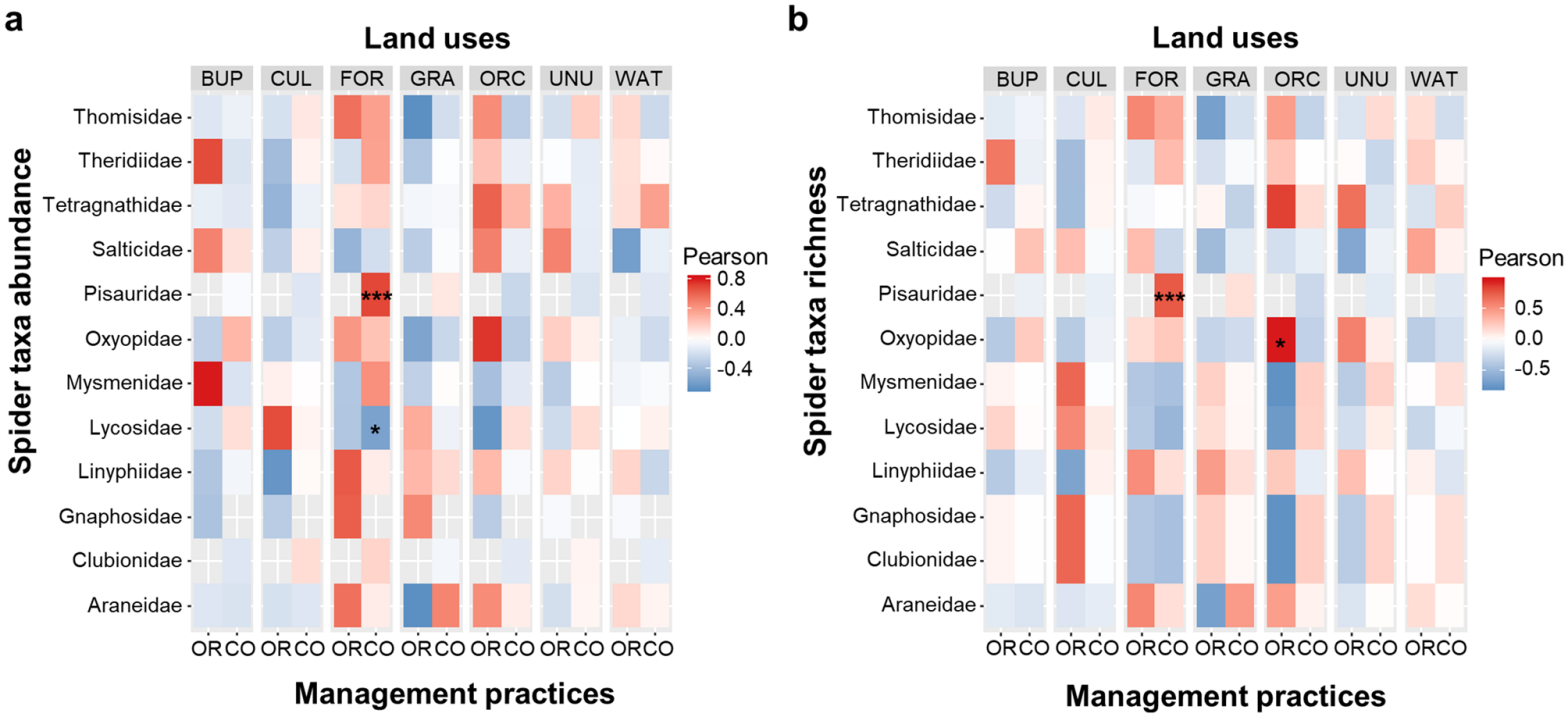
The relationship of spider (**a**) taxa abundance and (**b**) species richness with the proportions of different land use variables (“BUP” = built-up, “CUL” = cultivated, “FOR” = forest, “GRA” = grassland, “ORC” = orchard, “UNU” = unused, “WAT” = water) in the landscape based on correlation at levels of management practices (organic “OR” and conventional “CO”). The abundance and species richness of each spider taxa is correlated with each of the environmental variables. “*” is indicating the significant correlation.

**Figure 3:**
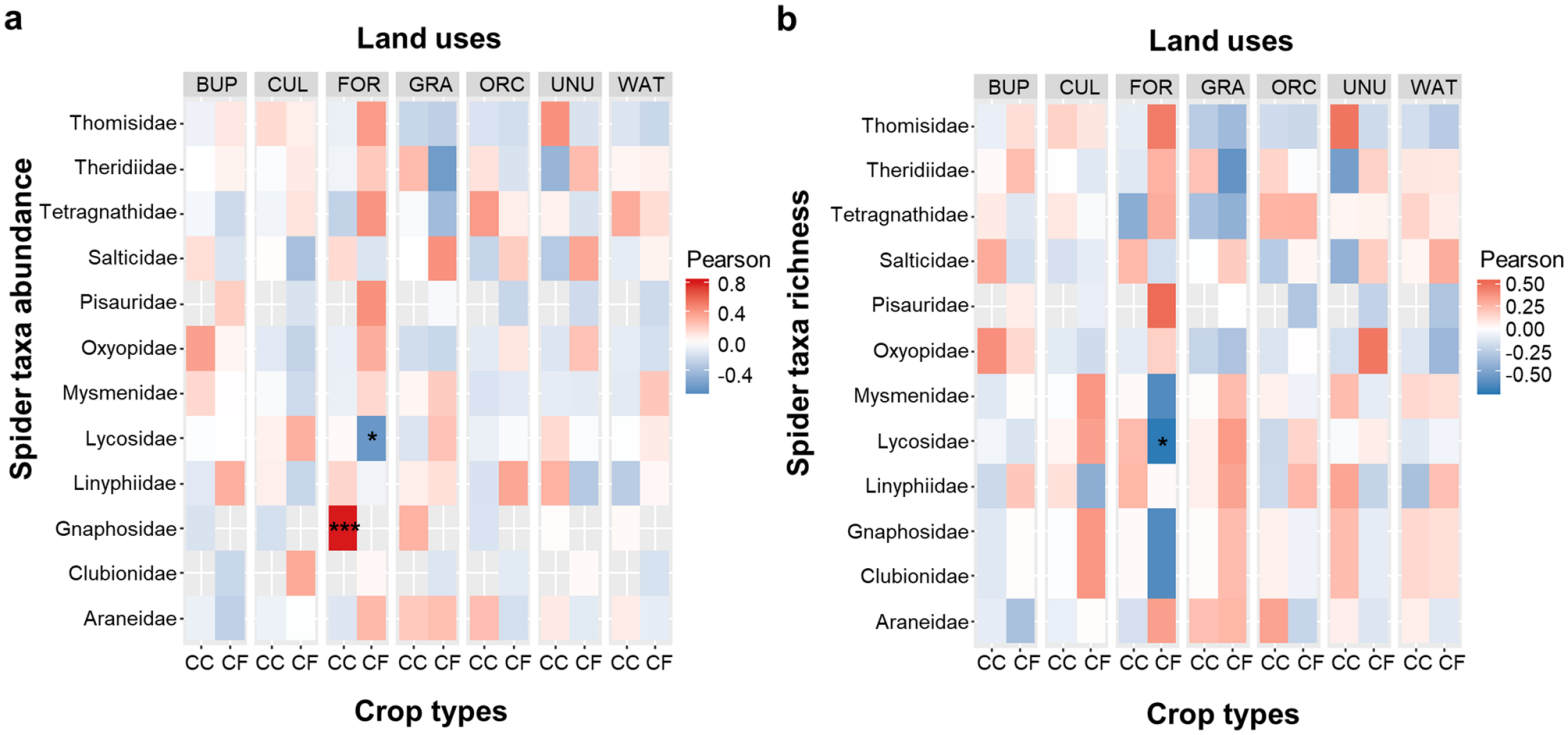
The relationship of spider (**a**) taxa abundance and (**b**) species richness with the proportions of different land use variables (“BUP” = built-up, “CUL” = cultivated, “FOR” = forest, “GRA” = grassland, “ORC” = orchard, “UNU” = unused, “WAT” = water) in the landscape based on correlation at levels of Brassica crop types (Chinese cabbage “CC” and cauliflower “CF”). The abundance and species richness of each spider taxa is correlated with each of the environmental variables. “*” is indicating the significant correlation.

Abundance and species richness of spider families were affected differently by local management and land use proportions in the landscape (Figs. 2, 3). Conventional fields with a higher proportion of forest in the surrounding landscape showed a positively significant relationship with the abundance and species richness of Pisauridae (Fig. 2a,b). On the other hand, statistically significant negative relationship of conventional fields with a higher proportion of forest in the surrounding landscape were observed with the abundance of Lycosidae (Fig. 2a,b). Moreover, the higher proportion of orchards around organic fields showed a significantly positive relationship with the richness of Oxyopidae (Fig. 2b).

Cauliflower fields surrounded with a higher proportion of forested land showed significantly negative correlation with the abundance and species richness of Lycosidae (Fig. 3a,b). In contrast, however, Chinese cabbage fields with a higher proportion of forests in the landscape were dominated by Gnaphosidae in terms of abundance only (Fig. 3a).

CCA models illustrated the response of spider families assemblages in terms of both abundance and species richness to different explanatory variables (including local factors and proportion of different landuses in surrounding landscape). In CCA models only the “built-up” landscape element was found to be redundant (have VIF > 10), so it was removed from the final CCA models. The CCA models explained 32% of total variability in the assemblages of spider families abundance and 34% of total variability in the assemblages of species in spider families. The results showed that the first three, out of the total 8, constrained eigenvalues (including management practices, crop type and proportion of forests in the landscape) accumulatively explained 82% of total variability in the assemblages of spider families abundance and 91% in the assemblages of species structure in spiders taxa (Fig. S1). In contrast, only small fractions of variability in assemblage structure of abundance and species richness in spider families were explained by the proportions of other land-uses (such as cultivated, grassland, unused, water and orchards).

An overall CCA model test of significance after 999 permutations of residuals showed that the canonical relationship of both the assemblages of abundance and richness in spider families with environmental predictors (including management practices, crop type and proportions of different land-uses in the landscape) was significant (*χ*^2^ = 0.247, F = 1.525, *p* = 0.013 and *χ*^2^ = 0.158, F = 1.676, *p* = 0.006, respectively) (Table 1). Additionally, during the permutations test, the management practices, crop type and proportion of forest land in the landscape were found to be significant in influencing the variations in the assemblage structure of abundance (*χ*^2^ = 0.057, F = 2.803, *p* = 0.010; *χ*^2^ = 0.068, F = 3.354, *p* = 0.002 and *χ*^2^ = 0.050, F = 2.667, *p* = 0.014, respectively) and species richness (*χ*^2^ = 0.023, F = 1.943, *p* = 0.054; *χ*^2^ = 0.055, F = 4.647, *p* = 0.001 and *χ*^2^ = 0.025, F = 2.143, *p* = 0.042, respectively) in spider families. However, proportion of grassland in the landscape significantly explained the variations in only the assemblage of species in spider families (*χ*^2^ = 0.030, F = 2.568, *p* = 0.021). The assemblage structure of both abundance and species richness in spider families was not significantly influenced by none of the other land uses in the landscape (including cultivated, unused, water, built-up and orchard) (Table 1).

**Table 1:** Permutation test for Constrained Correspondence Analysis (CCA) of spider abundance and richness in Brassica crop types (cauliflower or Chinese cabbage) managed under different management practices (conventional or organic) in sites with varying proportions of different land-uses.

Factors	Abundance			Richness		
	Chi-square	F-value	Pr(> F)	Chi-square	F-value	Pr(> F)
Management practices	0.057	2.803	0.010**	0.023	1.943	0.050*
Crop types	0.068	3.354	0.002**	0.055	4.647	0.001***
**Land-uses**						
Forest	0.054	2.667	0.014*	0.025	2.143	0.042*
Cultivated	0.007	0.346	0.968	0.003	0.281	0.970
Grassland	0.024	1.189	0.277	0.030	2.568	0.021*
Unsued	0.007	0.362	0.961	0.009	0.744	0.641
Water	0.015	0.765	0.591	0.006	0.518	0.832
Orchard	0.014	0.712	0.670	0.007	0.562	0.806
CCA model	0.247	1.525	0.013*	0.158	1.676	0.006*

The significance of constraint variables were tested by performing 999 permutations, “*”, “**” and “***” is indicating the significant constraints.

**Figure 4:**
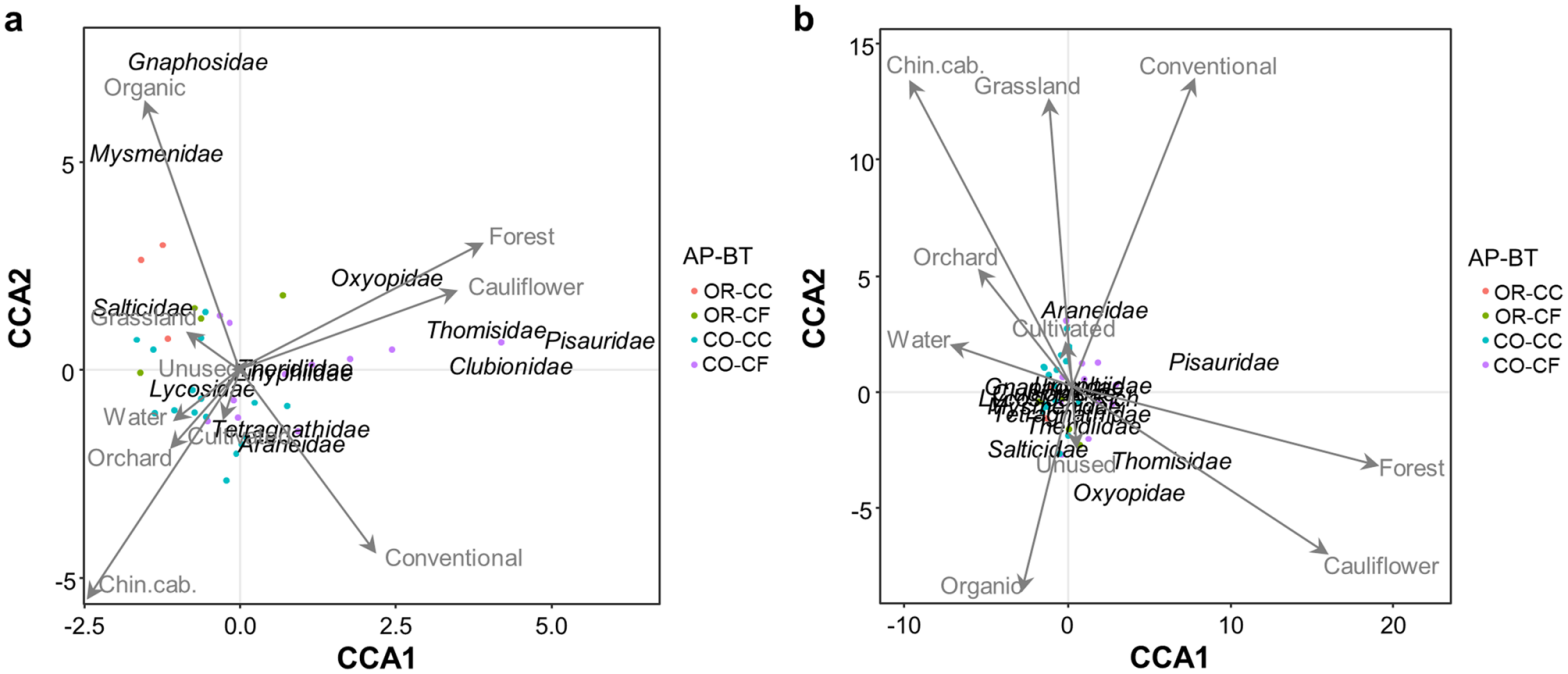
CCA ordination diagram with type II scaling represents the association of (**a**) abundance and (**b**) richness of 12 spider families found in Brassica crop types (cauliflower and “Chin.cab.” = Chinese cabbage) grown under different management practices (organic and conventional) across varying proportions of land-use variables. The arrow length and direction represent the magnitude of variance that can be explained by the explanatory and response variables. The perpendicular distance between spider families and explanatory variables reflects their correlations (below-90° = positive correlation and above-90° = negative correlation). The smaller the perpendicular distance, the stronger the correlation.

The CCA ordination results indicated that among local scale factors the organic versus conventional fields and cauliflower versus Chinese cabbage were separated on the first and second CCA axes (Fig. 4a,b). However, the landscape scale factors, the forested land was clustered along the first CCA axis. Whilst all other landscape variables were separated by the second CCA axis (Fig. 4a,b). The ordination showed positive response of most spider families (except Lycosidae, Salticidae, Mysmenidae and Gnaphosidae) in terms of abundance and species richness with organic fields, cauliflower crops and proportion of forest area in landscape (Fig. 4a,b). The abundance of Salticidae, Mysmenidae and Gnaphosidae positively responded with the organic practices and proportion of grassland in the landscape (Fig. 4a). Similarly, the abundance of Lycosidae responded positively to Chinese cabbage crops, and proportions of cultivated land, water and orchards in the landscape (Fig. 4a). The abundance and species richness of Araneidae strongly associated with the conventional fields (Fig. 4a,b).

## Discussion

Local scale management practices such as crop identity and chemical use, had direct effects on the assemblage structure of spider communities in highly disturbed and ephemeral vegetable growing systems, both in terms of abundance and species composition. Likewise, at a landscape scale, we found significant effects of the proportions of non-crop habitats (including forests and orchards) in the adjacent landscape. Other studies of less disturbed and persistent agroecosystems^15,28^ has also reported that assemblages of spider communities were significantly affected by both the farming systems and high percentage of non-crop habitats in the landscape. Studies have confirmed the persistent effects of local management practices on natural enemy diversity and their biological control potential^5^, and over wider landscape scale managing spatial distribution of both crop and non-crop habitats can be used as mean to reduce the pest population^12^. Although, purely based on landscape variables, the meta-analysis by Karp et al*.*^20^ found inconsistent responses of pests and predators to the surrounding landscape composition. The authors, however, suggested that local management factors should be incorporated in landscape models to further develop the accuracy of pest control systems. The results of the present study provide an evidence for the value of that suggestion, by highlighting the importance of crop species and management practices, as local factors, as well as the presence of non-crop habitats in the surrounding landscape.

Our results showed the influence of different crop types interact with surrounding landscape structure to differently drive the assemblage patterns of each spider families. This may be due to the differences in the functioning of crops, how efficiently they provide resources (such as food, shelter and prey items), for every spider family at a local field scale^30,31^. Moreover, previous studies of woodlands^32,33^ have reported that the density and diversity of spider assemblages are closely related to the structural composition and stability of the local habitat; because the enhancement in the structural complexity and stability of the habitats increased the density and diversity of predators by increasing their required resources (mainly food and shelter)^31,34^. The results of this study confirmed these findings; in a highly disturbed and ephemeral agroecosystem, the assemblage structure and composition of most spider families were positively responded with the cauliflower and presence of seminatural habitats in the surrounding landscape. This is likely to be due to the increased availability of heterogeneous micro-sites within the crop canopy of a complex plant structure, relatively more stable habitat because of long crop duration of cauliflower than Chinese cabbage for colonization/recolonization^35^. In addition to the heterogeneous micro-sites and stable habitat, the nearby seminatural habitat patches may provide more resources for spiders with diverse foraging behavior; such as web-builders (e.g., Pisauridae, Linyphiidae and Theridiidae), ambushers (Thomisidae), stalkers (Oxyopidae) and foliage runners (Clubionidae). In another study, Gangurde^36^, have reported the highest diversity and richness of predator species during the later growth stages of rice than the early growth stages. On the other hand, Chinese cabbage crop and low proportion of non-crop habitats in the landscape were correlated with the colonization of running spiders (Lycosidae). In this case, simple plant structure, small leaf surface area and less stable habitat because of the short Chinese cabbage crop duration for colonization/recolonization and that the low proportion of non-crop habitat patches in the surrounding landscape make the environment more suitable for the active hunters and likely to be unsuitable for the assemblages of diverse spider communities. The strong negative influence of Chinese crop growing systems on spider assemblages may be due to the lack of places for hiding and attaching their nets^33^, fewer prey items and high frequency of disturbance. The eminent dominance of Lycosids and lower number of web spiders in this highly dynamic brassica growing system may be because Lycosids have a high potential as early colonizers^35^ with high dispersal ability, whilst frequent disturbances (due to harvesting and intensive farming practices) does not favor the web spiders. In another study by Öberg and Ekbom^37^ sowing event was a detrimental factor for occurring of most of the spider families, but Lycosids as a group were not affected by the sowing events and were uniformly distributed in the field which may because of their high resilience to the environmental changes. In our study we reported the positive association of Lycosids with lesser non-crop habitat than those in with higher non-crop area. Similar patterns were also reported by Öberg^38^ where *Pardosa* spider was more dominant in the structurally simpler landscape than in structurally complex landscape. Altogether, these results highlighted the importance of crop species, crop duration and availability of non-crop habitats in the surrounding landscape to support the diverse spider assemblages at local field scale, which, in turn, can mediate the ecosystem service of pest suppression^24^.

In line with previous studies^10,15^ of less disturbed and persistent agroecosystems; results of our study of highly disturbed and ephemeral agroecosystem showed that local management practices interact differently with different land use in the landscape to determine the assemblage patterns of each spider family. The different interactions of local management practices with different land use may because of differences of local habitat quality^39^, the availability of prey items^15^ and frequency of disturbances between organic and conventional management practices. The results of this study indicated that the presence of non-crop habitats, especially woodlands and orchards, adjacent to the organic fields positively supported the diverse and abundant assemblages of spiders. Also, the management practices applied in organic fields promoting denser and more diverse vegetation^40^ are likely to enhance the availability and diversity of resources, which in turn, increase the density and species composition of spiders^41^. A recent study of vineyards by Muneret et al*.*^29^ highlighted that the deployment of organic practices at landscape scale significantly enhances the density and diversity of natural enemies, reduces the pest infestation and increases the productivity of the crop. On the other hand, synthetic pesticides used to causes rapid, detrimental effects on local fauna^5^ and our results have shown the impoverished assemblages of both abundance and richness in most spider families under conventional management practices surrounded with other land uses than non-crop habitat in the landscape, which may because the non-crop patches may act as nearby refuge or donor habitat for spiders during the period of disturbances^19^. However, conventional fields with lower proportion of non-crop habitat in the surrounding landscape were mainly dominated by the active hunters (Lycosidae), orb-weavers (Araneidae and Tetragnathidae). As Tetragnathidae and Araneidae spiders are commonly known as leaf curling and subterranean spiders, so their living characteristics may help them to avoid the high level of disturbance events by seeking shelter and nesting in the curled leaves and subterranean habitats. Similarly, Lycosids are active hunters and are also known as cursorial spiders (ground-runners), which are capable to quickly seek shelter during the disturbance regimes and actively forage over a large area in conventionally managed fields. The conventional fields surrounded with high proportion of forests also had significant correlation with another large and robust spider, like the Lycosids, known as roaming hunters (Pisauridae).

The results of this study highlighted a clear and direct influence of local factors on assemblage patterns of spiders both in terms of abundance and species composition. However, we also found support for the equivalent effects of patches of non-crop habitats in the surrounding landscape which has direct effects and substantially interacts with local factors to drive the response of generalist predator in a highly dynamic brassica agroecosystem. Several other studies showed that the occurrence of non-crop habitats drive the assemblages of spiders in an agroecosystem, both individually^14^ or by differently interacting with agricultural operation such as fertilizers and pesticides application^42^. This is because the patches of non-crop habitats in the surrounding landscape can supply a variety of alternative resources for a diverse group of predators including overwintering sites, shelter, refuge during the disturbance periods and food resources^31^, and as well as by favoring the dispersal of predators in the landscape matrix^43^.

In landscape studies, choosing the right spatial scale may influence the sensitivity of the method. A number of studies on how landscape elements at different spatial scales influence the spiders dispersal shape their assemblages supported that a relatively small study radius is sufficient to detect the influence of the surrounding landscape^44,45^. In our study, the effects of non-crop habitat on the abundance and species richness of most spider families at this smaller spatial scale were evident in this highly dynamic Brassica growing system. Correspondingly, the previous study of winter oilseed rape growing system by Drapela et al*.*^45^ also indicated that species richness of spiders was positively related to non-crop areas and woody areas at the smallest spatial scale of (250–500 m radius). Moreover, the study by Schmidt et al*.*^44^ in winter wheat indicated the contrasting response of different spider species (Lycosidae, Tetragnathidae and Thomisidae) at the different spatial scale ranging from 95 to 3,000 m, but the species richness of arable spiders were positively affected by the availability of heterogeneous landscape with the higher percentage of non-crop habitat at all spatial scales. Overall, the results of this study and previous studies indicated that different farmland spider species benefited differently from different land-use types in different growing systems. Still, the positive response of most spider families demonstrates the importance of non-crop habitats as a source of immigrants to the brassica fields at smaller spatial scales. In contrast to the previous studies, the sampling sites in this study belong to the smallholder farmers, and their agriculture operations (including high inputs of chemicals and destruction of natural habitat patches) pose severe threats to biodiversity^46^. Therefore, we need an integrated landscape approach with high opportunities for achieving long-term biodiversity conservation in smallholder farmlands at the smaller landscape scale. Thus, the results of this smaller landscape scale and previously studied large landscape scales clearly indicated that incorporation/conservation of non-crop habitats in the smallholder farms could provide buffer zones, food and shelter to the arable spiders.

During the last few decades, severe loss of biodiversity^47^ has driven agroecological researchers to consider interventions for more sustainable crop production system in which ecosystem services are promoted^7,8,12,20,39^. Our analysis of spider assemblages in a highly dynamic vegetable production systems shows that local factors and the patches of non-crop habitats in the surrounding landscape individually or substantially interact to drive the assemblage patterns of this important taxon of natural enemies. The net result of these changes in assemblage patterns of spiders is likely to affect the strength of top-down pest suppression by these important ecosystem providers. This study suggested that the inconsistencies in responses of predators to the surrounding landscape composition, as highlighted by Karp et al.^20^, were potentially caused by local factors, including chemical use and type of crops. The population structure of generalist predators in this study provides a solid foundation for future studies to determine the relative extent to which local factors and different landscape variables influence the relative strength of their ecosystem function. Efforts in this direction will provide important strategic guidance to farmers and policymakers for how best to allocate effort to interventions at differing spatial scales in the pursuit of sustainable ecological intensification.

## Methods

### Study region and site selection

**Figure 5:**
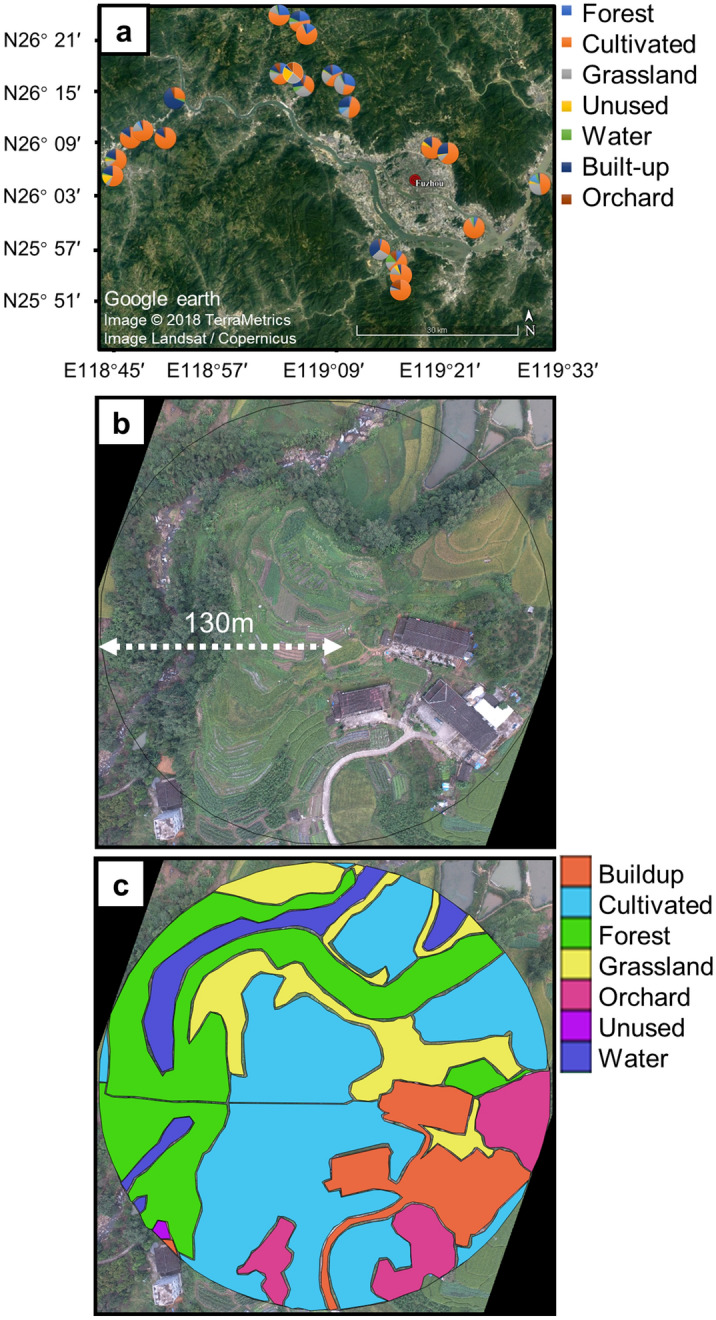
(**a**) Locations of focal brassica fields in the region of Fuzhou City, Fujian province, China (image obtained from Google satellite map using Google earth software https://earth.google.com/web/). Pies show the composition of the (**b**) landscape at 130 m radius around the focal fields. (**c**) Mapping of drone-based georeferenced (using QGIS https://qgis.osgeo.org) high-resolution image of a focal sampling site in the region of Fuzhou City, Fujian province, China.

All experimental protocols were carried out in strict compliance with the State Key Laboratory of Ecological Pest Control for Fujian and Taiwan Crops and were approved by the ethics committee of Fujian Agriculture and Forestry University. The informed consent was obtained to assure that the farmers know every aspect of their participation. Field sites were located in Fujian Province, southeastern China, and data were collected during the main Brassica growing season (August–November) in 2017. The Fujian province in china is mostly mountainous and it has a subtropical, warm and humid, climate. Typical farming systems here are smallholders of highly dynamic Brassica crops that are globally common in other agricultural systems. The region’s average temperature is 7–10 °C in mid-winter, while the average temperature is 23–33 °C at the peak of summer. Fujian has an annual precipitation of 1,400–2,000 mm. Fields were 1,300–2,000 m^2^ in size which is typical for the region and were selected to represent different management practices and crop types with various proportions of land uses in the surrounding landscapes. The 23 fields were marked out using GPS (GPSMAP 60CSx-Garmin) and were at least 1 km apart to minimize the influence of spatial autocorrelation (Fig. 5a).

Fields were allocated into two groups, according to management practices: seventeen conventionally managed (i.e., synthetic pesticides or fertilizers applied) and six Chinese government-certified organics (no synthetic chemical inputs). The uneven number of fields in these two groups reflected their relative representation in this region (Table S1). Statistically adequate replicates of cauliflower (*Brassica oleracea*) and Chinese cabbage (*Brassica rapa pekinensis*) crop types were represented among both organic and conventional management. Direct sowing and seedling transplanting cropping methods were used to grow Chinese cabbage and cauliflower, respectively. Chinese cabbage is known as small head Brassica cultivar, so Chinese cabbage seeds directly sowed on the soil beds of approximately sized ~ 600–900 mm. However, for cauliflower, known as large head Brassica cultivar, seedlings were grown first, and then two weeks after sowing seedlings were transplanted to the field on both edges of soil beds (sized ~ 600–900 mm) with the plant to plant distance ~ 150 mm.

To map the surrounding landscape structure, aerial images were taken using an unmanned aerial vehicle (UAV) (*DJI*-PHANTOM 4, Shenzhen Dajiang Baiwang Technology Co., Ltd., China). The proportions of different land use in the surrounding landscape were estimated at a spatial scale of 130 m radius, which was sufficient radius for this landscape scale study of spiders^44^, using QGIS software (version-2.18.27) at each site (Fig. 5b). A total of seven land uses in the surrounding landscape were quantified (Fig. 5c): forest (naturally grown trees or non-fruit tree plantations), cultivated (tillage land, annual and perennial crops), grassland (artificial grassland, natural grassland, hedgerows and shrubs), unused (barren land or empty), water (river, pond, irrigation channel and reservoir), built-up (road, residential land, greenhouse and other built-ups) and orchard (loquat, litchi, citrus and other fruit trees). The proportional composition of different land uses in the landscape were calculated at 130 m radius: forest (5.57% ± 1.41% (mean SEM throughout the text), range 0.00–26.09%), cultivated (64.66% ± 3.09%, range 20.70–83.30%), grassland (12.82% ± 1.44%, range 0.00–29.06%), unused (3.25% ± 1.03%, range 0.00–23.84%), water (3.67% ± 0.76%, range 0.00–16.83%), built-up (16.63% ± 2.59%, range 1.87–64.82%) and orchard (3.40% ± 0.87%, range 0.00–17.06%) (see Table S1 and Fig. S2 for complete details).

### Sampling and identification of spiders

Spiders were collected from each site following a sampling method 4 given in the Sørensen et al*.*^48^ and Mader et al*.*^49^ due to the suitability and less chances of damaging the leafy Brassica crops. In each brassica field, spiders were collected individually by visually searching the soil surface and plants for one hour by two persons. A total of 35 sampling visits was performed over 23 sampling sites. Spiders were placed individually in vials, transferred to an ice box for transportation to the laboratory and thereafter kept at − 80 °C. All individuals were photographed and were assigned to a morphospecies. Later, molecular identification was used to determine the actual species of visually sorted morphospecies (see below).

### Molecular identification of morphospecies

Genomic DNA was extracted from an excised leg from a representative specimen of each visually assigned morphospecies using Qiagen DNeasy blood and tissue kit (Qiagen Inc., USA) following the manufacturer’s instructions. Prior to DNA extraction, each specimen was surface sterilized using 100% ethanol and then rinsed with purified water. Polymerase chain reaction (PCR) was performed to amplify the ~ 658 bp region of mitochondrial COI gene using well established universal primer pair for arthropods (e.g., Forward-LCO1490 *5′-GGTCAACAAATCATAAAGATATTGG-3′* and Reverse-ChelicerateReverse2 *5′-GGATGGCCAAAAAATCAAAATAAATG-3′*). PCR reaction contained the 1 × Hieff PCR Master Mix, 2 µl of template DNA, 1 µl of each primer pair (10 M) and 8.5 µl of nuclease free water to make a total volume of 25 µl. PCR programming was set as follows; initial denaturation at 94 °C for 120 s, followed by 35 cycles of 94 °C for 45 s, 48 °C for 45 s and 72 °C for 30 s. The final extension was performed at 72 °C for 300 s. Gel electrophoresis was performed to verify the successful amplification of target DNA fragments. PCR products were stored at 4 °C before send to BioSune sequencing facility located in Shanghai. Contig sequences were annotated for each amplified DNA of spiders based on similarity with available sequences in GeneBank (https://www.ncbi.nlm.nih.gov/) to identify the each visually sorted morphospecies (see Supplementary dataset 2). All the DNA barcodes were submitted to NCBI Genebank with accession numbers from MF467584 to MF467725 (https://www.ncbi.nlm.nih.gov/). One voucher specimen of each morphospecies was lodged in the Museum of Fujian Agriculture and Forestry University, Fujian, China.

### Data analysis

Alpha diversity (diversity among samples) of the spider community data was measured using richness, Simpson and Shannon indices. Kruskal–Wallis rank sum test for each selected alpha diversity index was performed to identify the significance of variations between management practices and crop types^50^.

Due to the high proportion of rare species in our samples, instead of using species census data, we calculated the most widely used community measures, abundance and species richness, for each spider family. Community matrices were then populated with these, per family abundance or species richness, data, and were further analyzed using the “vegan” package^51^ in R. Pearson correlation test was performed to show the relationship of spider abundance, and species richness with the selected environmental variables (management practices, crop types and different land-use in the surrounding landscape). Moreover, the associated *p*-values were also calculated to show the significance of the relationships and adjusted following the Benjamin and Hochberg procedure^52^. Correlation heatmaps were drawn by following the R-codes provided by Torondel et al.^53^.

Canonical correspondence analysis (CCA) was used to investigate how environmental variables can differently influence the assemblage structure (in terms of both abundance and species richness) of spider communities. Our community matrices, with the abundance and species richness data, of spider families were Hellinger transformed prior to analysis, since this transformation enables the use of ordination methods along with Euclidean distances based on datasets containing many zeros^54^. Variance Inflation Factors (VIF) for each of the environmental variables were calculated to test the goodness of fit in our CCA model. Since environmental variables with VIF > 10 present collinearity with other environmental variables^55^, and they do not contribute to explaining the variance in the model significantly, these were removed from our final model. An ANOVA-like permutation test was performed for testing the significance of the CCA model and environmental variables^56^. Likewise, the differences between spider communities, based on both abundance and species richness, were then statistically tested using the analysis of similarity (ANOSIM) method^57^.

All calculations were conducted, and graphs were drawn in R, using the “BiodiversityR”^58^, ‘‘vegan’’^51^, ‘‘gplots’’^59^, “Heatplus”^60^ packages and following the R-codes provided by Torondel et al.^53^. We do, however, acknowledge that the sampling method we used, like all sampling methods, can be biased so lead to some taxa (such as those that are highly cryptic) appearing rarer in samples than in the biological community.

## Supplementary information

Supplementary Information 1.

Supplementary Information 2.

## Data Availability

All data sheets relative to the tests run are available as supplementary materials.
