# Supplementary material for: Local management and landscape structure determine the assemblage patterns of spiders in vegetable fields
Source: Sci Rep. 2020 Sep 15;10:15130. doi: 10.1038/s41598-020-71888-w (PMC7493935; doi:10.1038/s41598-020-71888-w)
Supplement: Supplementary file 1 — Supplementary Information 1. [file 41598_2020_71888_MOESM1_ESM.docx]

Supplementary data

# **Local management and landscape structure determine the assemblage patterns of spiders in vegetable fields**

Hafiz Sohaib Ahmed Saqib^1,2,3^, Junhui Chen^1,2,3^, Wei Chen^1,2,3^, Gabor Pozsgai^1,2,3^, Komivi Senyo Akutse^5^, Muhammad Furqan Ashraf^6^, Minsheng You^1,2,3,4*^ and Geoff M. Gurr^1,2,3,7*^

^1^ State Key Laboratory of Ecological Pest Control for Fujian and Taiwan Crops, Fujian Agriculture and Forestry University, Fuzhou 350002, China

^2^ Joint International Research Laboratory of Ecological Pest Control, Ministry of Education, Fuzhou 350002, China

^3^ Institute of Applied Ecology, Fujian Agriculture and Forestry, University, Fuzhou 350002, China

^4^ Key Laboratory of Integrated Pest Management for Fujian-Taiwan Crops, Ministry of Agriculture, Fuzhou 350002, China

^5^ Plant Health Division, International Centre of Insect Physiology and Ecology (ICIPE), P.O. Box 30772-00100, Nairobi, Kenya.

^6^ College of Crop Science, Fujian Agriculture and Forestry, University, Fuzhou 350002, China

^7^ Graham Centre, Charles Sturt University, Orange, NSW 2800, Australia

^*^ Corresponding authors e-mail: [ggurr@csu.edu.au](mailto:ggurr@csu.edu.au); [msyou@fafu.edu.cn](mailto:msyou@fafu.edu.cn)

**Table S1** Information corresponds to the focal field locations, Brassica crop species (“CF” cauliflower and “CC” Chinese cabbage), agronomic practices (organic and conventional) and percentage proportion of various land-used present in 130m radius of the sampling field.

| **Sample** | **Latitude** | **Longitude** | **Agrono. practices** | **Brass. species** | **Forest** | **Cultivated** | **Grassland** | **Unsued** | **Water** | **Built-up** | **Orchard** |
| --- | --- | --- | --- | --- | --- | --- | --- | --- | --- | --- | --- |
| Shengzhiyang | 26° 14' 54.63" | 119° 04' 54.43" | Organic | CF | 0.00 | 36.24 | 23.80 | 23.84 | 0.00 | 5.16 | 10.96 |
| Changle | 26° 03' 45.97" | 119° 31' 38.95" | Organic | CF | 0.00 | 72.14 | 19.70 | 0.00 | 4.91 | 3.24 | 0.00 |
| Kongyuan | 26° 14' 25.74" | 119° 5' 45.93" | Organic | CC | 9.19 | 39.55 | 22.54 | 0.00 | 8.23 | 19.26 | 1.22 |
| Kongyuan | 26° 14' 25.74" | 119° 5' 45.93" | Organic | CF | 9.19 | 39.55 | 22.54 | 0.00 | 8.23 | 19.26 | 1.22 |
| Weicheng | 26° 15' 42.95" | 119° 3' 14.06" | Organic | CF | 24.49 | 39.79 | 12.26 | 0.14 | 5.81 | 9.85 | 7.67 |
| Xiaomifeng | 26° 14' 25.33" | 119° 10' 22.27" | Organic | CC | 26.09 | 36.11 | 25.52 | 3.21 | 4.41 | 4.66 | 0.00 |
| Guxiang | 26° 12' 9.13" | 119° 10' 46.77" | Organic | CC | 0.00 | 52.22 | 13.97 | 0.00 | 0.95 | 24.39 | 0.00 |
| Baizhong | 26° 7' 53.81" | 118° 43' 49.11" | Conven. | CC | 0.00 | 56.21 | 5.78 | 16.58 | 1.77 | 19.66 | 0.00 |
| Bandong | 26° 7' 46.36" | 118° 44' 35.25" | Conven. | CC | 0.00 | 48.90 | 24.77 | 3.13 | 4.55 | 18.64 | 0.00 |
| Nantong4 | 25° 55' 29.06" | 119° 15' 43.73" | Conven. | CC | 0.00 | 67.67 | 9.83 | 0.00 | 0.30 | 5.13 | 17.06 |
| Nantong4 | 25° 55' 29.06" | 119° 15' 43.73" | Conven. | CF | 0.00 | 67.67 | 9.83 | 0.00 | 0.30 | 5.13 | 17.06 |
| Rongyuan | 26° 15' 19.44" | 119° 5' 41.13" | Conven. | CF | 11.92 | 44.71 | 25.26 | 0.00 | 1.44 | 14.50 | 2.17 |
| Bandong | 26° 7' 46.36" | 118° 44' 35.25" | Conven. | CF | 0.00 | 48.90 | 24.77 | 3.13 | 4.55 | 18.64 | 0.00 |
| Xiqian | 26° 09' 56.52" | 118° 46' 19.46" | Conven. | CF | 2.41 | 66.62 | 0.00 | 0.00 | 15.46 | 15.52 | 0.00 |
| Guanzhuang | 26° 09' 47.02" | 118° 50' 11.06" | Conven. | CC | 0.97 | 76.73 | 6.67 | 0.00 | 3.36 | 12.27 | 0.00 |
| Xinan | 26° 10' 48.51" | 118° 47' 26.48" | Conven. | CC | 0.00 | 79.87 | 5.21 | 5.81 | 0.00 | 2.56 | 1.95 |
| Baizhong | 26° 07' 53.81" | 118° 43' 49.1" | Conven. | CF | 0.00 | 56.21 | 5.78 | 16.58 | 1.77 | 19.66 | 0.00 |
| Dahuxiang2 | 26° 20' 47.02" | 119° 05' 37.27" | Conven. | CF | 19.31 | 45.92 | 12.02 | 0.00 | 1.95 | 20.80 | 0.00 |
| Dahuxiang1 | 26° 20' 50.1" | 119° 05' 00.45" | Conven. | CF | 21.04 | 57.71 | 11.37 | 3.20 | 2.74 | 3.95 | 0.00 |
| Dahuxiang1 | 26° 20' 50.1" | 119° 05' 00.45" | Conven. | CF | 21.04 | 57.71 | 11.37 | 3.20 | 2.74 | 3.95 | 0.00 |
| Dahuxiang2 | 26° 20' 47.02" | 119° 05' 37.27" | Conven. | CF | 19.31 | 45.92 | 12.02 | 0.00 | 1.95 | 20.80 | 0.00 |
| Dahuxiang3 | 26° 20' 15.14" | 119° 05' 33.72" | Conven. | CF | 10.89 | 70.32 | 8.74 | 4.29 | 0.68 | 5.07 | 0.00 |
| MinqingBA | 26° 13' 41.96" | 118° 51' 21.85" | Conven. | CC | 1.14 | 21.19 | 6.28 | 0.40 | 3.72 | 64.81 | 2.44 |
| Nantong1 | 25° 57' 31.20" | 119° 15' 19.25" | Conven. | CC | 5.33 | 20.70 | 29.06 | 0.00 | 1.81 | 33.69 | 9.42 |
| Chengmen | 25° 59' 44.56" | 119° 24' 08.87" | Conven. | CC | 0.00 | 83.30 | 6.80 | 0.00 | 3.70 | 1.87 | 4.33 |
| Nantong3 | 25° 55' 56.14" | 119° 15' 24.96" | Conven. | CC | 0.00 | 74.65 | 6.27 | 0.00 | 0.00 | 17.83 | 1.26 |
| Jiantian | 26° 07' 47.62" | 119° 19' 48.20" | Conven. | CC | 0.00 | 70.69 | 1.94 | 14.46 | 0.15 | 12.77 | 0.00 |
| Nantong2 | 25° 56' 40.61" | 119° 15' 23.17" | Conven. | CC | 0.00 | 53.59 | 15.14 | 0.00 | 16.83 | 2.08 | 12.36 |
| Jiantian | 26° 07' 47.62" | 119° 19' 48.20" | Conven. | CC | 0.00 | 70.69 | 1.94 | 14.46 | 0.15 | 12.77 | 0.00 |
| Nantong2 | 25° 56' 40.61" | 119° 15' 23.17" | Conven. | CC | 0.00 | 53.59 | 15.14 | 0.00 | 16.83 | 2.08 | 12.36 |
| Nantong3 | 25° 55' 56.14" | 119° 15' 24.96" | Conven. | CC | 0.00 | 74.65 | 6.27 | 0.00 | 0.00 | 17.83 | 1.26 |
| Chengmen | 25° 59' 44.56" | 119° 24' 08.87" | Conven. | CC | 0.00 | 83.30 | 6.80 | 0.00 | 3.70 | 1.87 | 4.33 |
| Nantong1 | 25° 57' 31.20" | 119° 15' 19.25" | Conven. | CC | 5.33 | 20.70 | 29.06 | 0.00 | 1.81 | 33.69 | 9.42 |
| Pudang | 26° 07' 50.51" | 119° 19' 56.55" | Conven. | CF | 6.29 | 58.05 | 3.94 | 0.91 | 0.00 | 30.81 | 0.00 |
| MinqingBA | 26° 13' 41.96" | 118° 51' 21.85" | Conven. | CC | 1.14 | 21.19 | 6.28 | 0.40 | 3.72 | 64.81 | 2.44 |


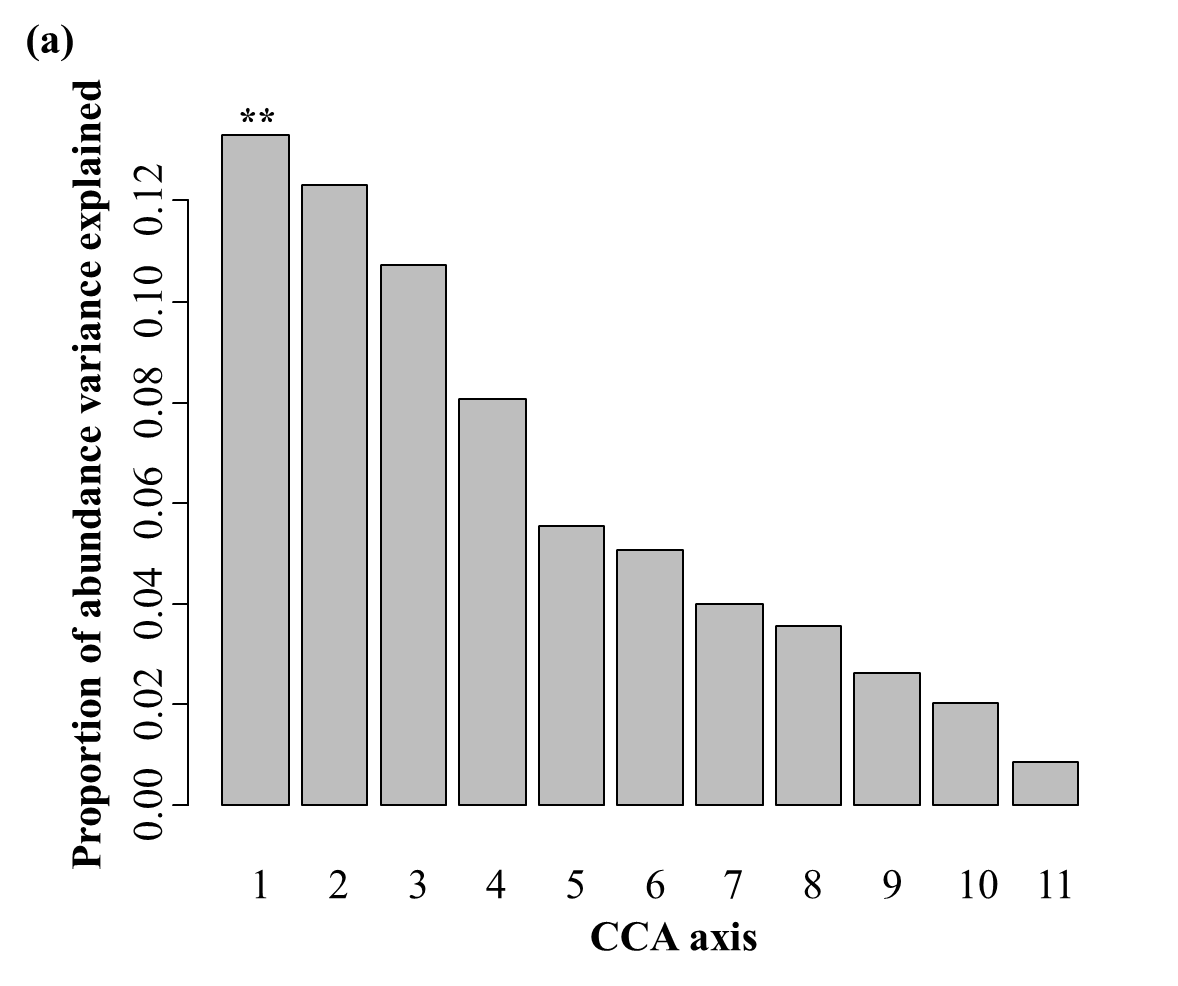


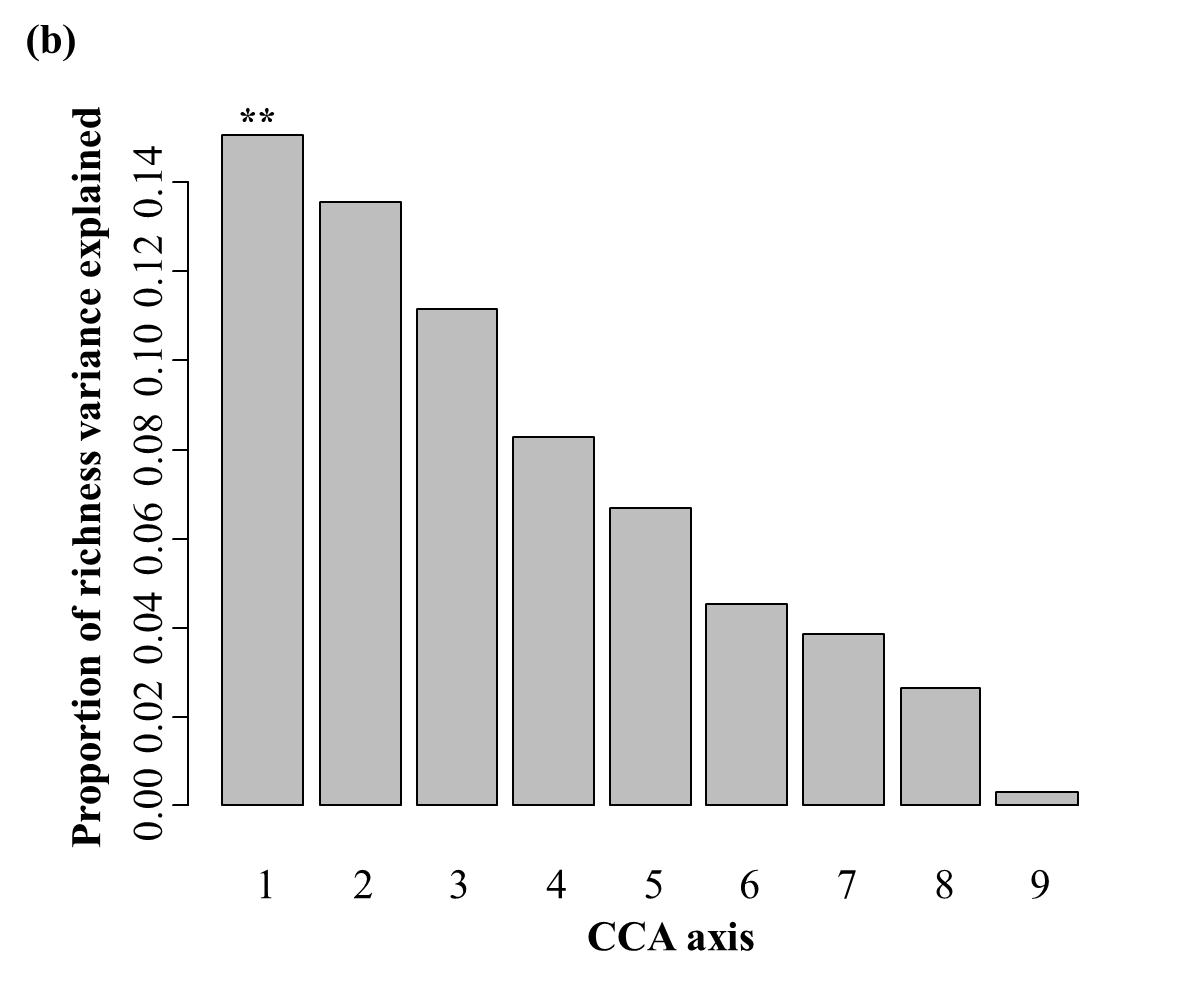


**Fig. S1** Barplot showing the proportion of variance in spider **(a)** abundance and **(b)** richness, explained by each of the CCA axis.


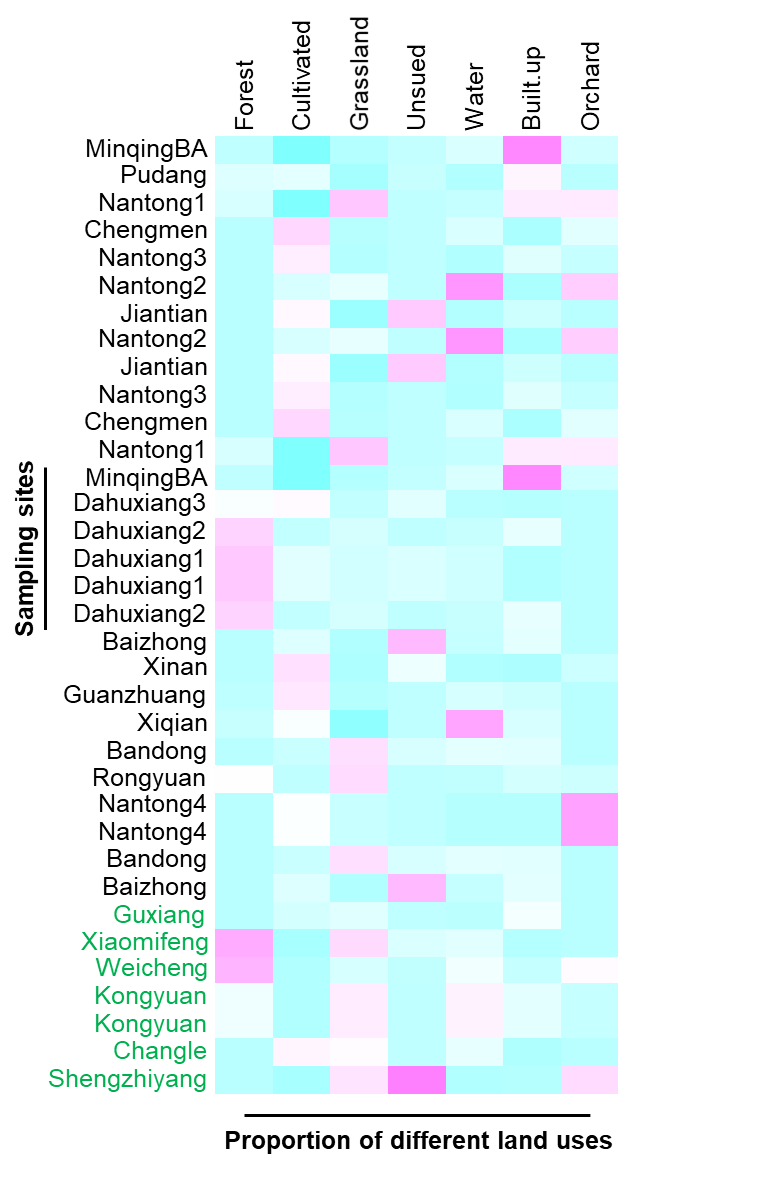


**Fig. S2** Heatmap showing the proportions of different land used in the surrounding landscape (radius ~130m) of all sampling sites. The names of sampling sites in green color represent the organic fields and black color represents the conventional fields. Heatmap was drawn using the “Heatplus” package in R software.
